# Rapid antimicrobial susceptibility testing of clinical isolates by digital time-lapse microscopy

**DOI:** 10.1007/s10096-015-2492-9

**Published:** 2015-09-25

**Authors:** M. Fredborg, F. S. Rosenvinge, E. Spillum, S. Kroghsbo, M. Wang, T. E. Sondergaard

**Affiliations:** Department of Animal Science, Faculty of Science and Technology, Aarhus University, Blichers Allé 20, 8830 Tjele, Denmark; Department of Clinical Microbiology, Vejle Hospital, Kabbeltoft 25, 7100 Vejle, Denmark; Philips BioCell, Gydevang 42, 3450 Allerød, Denmark; Department of Clinical Microbiology, Aarhus University Hospital, Palle Juul-Jensens Boulevard 99, 8200 Aarhus, Denmark; Department of Biotechnology, Chemistry and Environmental Engineering, Aalborg University, Sohngaardsholmsvej 49, 9000 Aalborg, Denmark

## Abstract

Rapid antimicrobial susceptibility testing (AST) is essential for early and appropriate therapy. Methods with short detection time enabling same-day treatment optimisation are highly favourable. In this study, we evaluated the potential of a digital time-lapse microscope system, the oCelloScope system, to perform rapid AST. The oCelloScope system demonstrated a very high accuracy (96 % overall agreement) when determining the resistance profiles of four reference strains, nine clinical isolates, including multi-drug-resistant isolates, and three positive blood cultures. AST of clinical isolates (168 antimicrobial agent–organism combinations) demonstrated 3.6 % minor, no major and 1.2 % very major errors of the oCelloScope system compared to conventional susceptibility testing, as well as a rapid and correct phenotypic detection of strains with methicillin-resistant *Staphylococcus aureus* (MRSA) and extended-spectrum β-lactamase (ESBL) profiles. The net average time-to-result was 108 min, with 95 % of the results being available within 180 min. In conclusion, this study strongly indicates that the oCelloScope system holds considerable potential as an accurate and sensitive AST method with short time-to-result, enabling same-day targeted antimicrobial therapy, facilitating antibiotic stewardship and better patient management. A full-scale validation of the oCelloScope system including more isolates is necessary to assess the impact of using it for AST.

## Introduction

Bacterial multidrug resistance is emerging worldwide at an alarming rate and is now recognised as a major public health threat [1]. This crisis is not likely to be solved by new antibiotics due to the low rate of antibiotic discovery and by the probability that pathogens will continue to evolve resistance to antibiotics. The initiation of effective antibiotic therapy early in the course of an infection may reduce the probability of pathogens evolving resistance [2]. Especially in the case of sepsis and bloodstream infections, early appropriate antimicrobial therapy is important to decrease mortality [3]. Sepsis management includes empirical antimicrobial therapy started as soon as possible without awaiting results from antimicrobial susceptibility testing (AST) [4, 5]. As empirical antimicrobial therapy is mainly composed of broad-spectrum antibiotics, this can perpetuate the cycle of increasing resistance [6]. Consequently, early determination of antimicrobial susceptibility is pivotal in targeted antimicrobial therapy in order to combat the escalating rates of resistance, as well as to decrease mortality.

Today, AST in routine clinical microbiology laboratories is generally performed by conventional methods such as disc diffusion, broth dilution or Etest [7, 8]. During the last decade, several technologies have been suggested as candidates for more accurate AST, e.g. whole-genome sequencing, mass spectrometry, fluorescence-activated cell sorting and microarrays [7]. Technologies relying on genotypic and proteomic analysis of resistance determinants such as whole-genome sequencing, polymerase chain reaction (PCR) and matrix-assisted laser desorption/ionisation time-of-flight (MALDI-TOF) have the key limitation in that they are unable to detect novel resistance mechanisms [9, 10]. In addition, some of these new techniques are time-consuming and characterised by expensive instruments, high analysis costs, the need for specialised technical personnel and are, in some cases, limited by single-sample analysis. Automated instruments such as Vitek 2 (bioMérieux), Phoenix 100 (BD Biosciences) and MicroScan WalkAway (Siemens) are commonly used methods for AST as they are easy to operate and reduce the time-to-result [11–14]. Nevertheless, in clinical practice, even faster AST methods are required if effective treatment with targeted antibiotic is to be achieved within the same working day.

In a previous study, we demonstrated the oCelloScope system to be a fast and sensitive, high-throughput AST method capable of detecting antibiotic susceptibility within 6 min for *Escherichia coli* and within 30 min in complex urine samples from pigs suffering from urinary tract infections [15]. In this study, we performed a preliminary evaluation of the ability of the oCelloScope system to analyse antimicrobial resistance by monitoring bacterial cell growth. The accuracy of the oCelloScope system was examined together with the time required for AST. This initial screening constitutes the proof of concept of the oCelloScope system in relation to AST of clinical isolates.

## Materials and methods

### Strains

Four quality control (QC) reference strains were included to validate the antimicrobial susceptibility results obtained by the oCelloScope system: *Escherichia coli* ATCC 25922, *Staphylococcus aureus* ATCC 29213, *Enterococcus faecalis* ATCC 29212 and *Streptococcus pneumoniae* ATCC 49619. The QC reference strains used are recommended for quality control by the Clinical and Laboratory Standards Institute (CLSI) and the European Committee on Antimicrobial Susceptibility Testing (EUCAST). In addition, nine clinical isolates, collected in Denmark during the period 2008–20012, were included: two *S. epidermidis* [one oxacillin resistant (*mecA*-positive) and one oxacillin and penicillin susceptible], one *S. pneumoniae* (penicillin intermediate and trimethoprim/sulphamethoxazole resistant), two *S. aureus* [one methicillin-resistant *S. aureus* (MRSA) (*mecA*-positive) and one methicillin-susceptible *S. aureus* (MSSA)], two *E. faecalis* [both gentamicin high-level resistant and one vancomycin resistant (*vanB*-positive)] and two *E. coli* [one extended-spectrum β-lactamase (ESBL) and one resistant to ampicillin, gentamicin and ciprofloxacin].

### Blood culture instrumentation and media

Blood samples from patients (8–10 mL/patient) with suspected bacteraemia were collected in standard aerobe non-charcoal BacT/ALERT FA blood culture bottles (bioMérieux, Marcy-l’Etoile, France) and handled according to the manufacturer’s instructions. Blood culture bottles were incubated in the BacT/ALERT 3D system (bioMérieux, Marcy-l’Etoile, France) at Aarhus University Hospital (Aarhus, Denmark). Blood cultures detected as positive by the BacT/ALERT 3D system were Gram stained and examined for bacterial morphology by light microscopy. All blood cultures used in this study were recognised as positive during the night and not processed until 8 o’clock the following morning. Species identification of isolates by the clinical laboratory was provided after the completion of oCelloScope AST measurements. Consequently, only preliminary phenotypic characterisation including Gram staining and examination of cellular morphology by light microscopy was provided prior to oCelloScope AST.

### Antimicrobial susceptibility testing by manually interpreted Sensititre® plates

AST was performed on all bacterial isolates using the Sensititre® system (TREK Diagnostic Systems, Cleveland, OH, USA), according to the manufacturer’s instructions. Three different Sensititre® plates were used: NF: *E. coli*, GPALLIF: Staphylococci and Enterococci, and STP6F: *S. pneumoniae*. Bacterial isolates were grown overnight on 5 % horse blood agar (Statens Serum Institut, Copenhagen, Denmark). Colony material was emulsified in sterile water (*E. coli*, *Staphylococcus* spp. and *Enterococcus* spp.) or Sensititre® cation-adjusted Mueller–Hinton broth with TES (CAMHBT, TREK Diagnostic Systems (*S. pneumoniae*) and the suspension was adjusted to 0.5 McFarland standard using the Vitek DensiChek densitometer (bioMérieux). For *Staphylococcus* spp., *Enterococcus* spp. and *E. coli*, 10 μL was transferred into 11 mL CAMHBT and 50 μL of the suspension was inoculated into each well on the Sensititre® plate. For *S. pneumoniae*, 100 μL was transferred into 11 mL Sensititre® cation-adjusted Mueller–Hinton broth with TES and lysed horse blood (CAMHBT + LHB, TREK Diagnostic Systems) and 100 μL of the suspension was inoculated into each well on the Sensititre® plate. Plates were incubated for 20–24 h at 35 °C. For each test, we performed an inoculum check according to the manufacturer’s instructions. AST results were read using the Sensititre® Manual viewer.

### Antimicrobial susceptibility testing by the oCelloScope system

QC reference strains and clinical isolates were grown under atmospheric conditions at 37 °C in CAMHBT, except *S. pneumoniae*, which was grown in CAMHBT + LHB. All isolates were grown overnight and transferred to new tubes and incubated for 2 h before AST. Positive blood cultures were prepared by centrifugation at 200 × *g* for 5 min (Sigma 3-18 k, 12171 rotor, Buch & Holm, Herlev, Denmark) to remove human blood cells, followed by resuspension of the bacterial pellet in CAMHBT. All broth–bacteria suspensions were diluted to McFarland 0.5 based on OD_600_ measurements using a UV-3100 PC Spectrophotometer (VWR, Herlev, Denmark) and subsequently diluted in cation-adjusted Mueller–Hinton broth to a final bacterial cell suspension of approximately 1 × 10^5^ bacteria/mL for all bacteria except for *S. pneumoniae*, which was diluted to a final concentration of approximately 5 × 10^5^ bacteria/mL. To facilitate focus adjustment of the oCelloScope system, beads were added to bacterial cell suspensions (2 × 10^4^ 6-μm beads/mL, microsphere standard, B7277, Invitrogen, Naerum, Denmark). Broth–bacteria–bead suspensions were added to Sensititre® plates (TREK Diagnostic Systems). NF, GPALL1F and STP6F plates were used as specified above. Bacterial cell suspensions were carefully transferred from the round-bottomed Sensititre® plates to flat-bottomed Nunc Edge 96-well plates (Thermo Fisher Scientific, Roskilde, Denmark) in order to be analysed by the oCelloScope system, as it cannot analyse round-bottomed plates. The determination of minimum inhibitory concentration (MIC) values for all experiments on QC reference strains and clinical isolates was carried out in triplicate experiments. The results were reported when at least two out of three MIC results were in agreement.

**Fig. 1:**
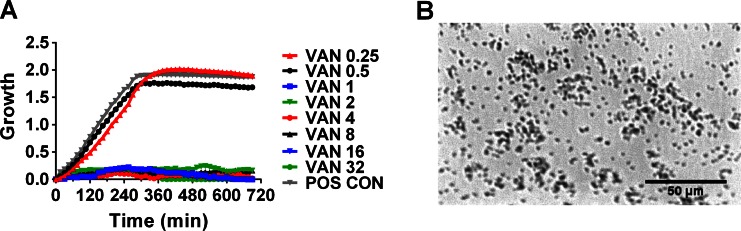
Example of an oCelloScope image and graphical output. **a** Growth curves for *S. aureus* ATCC29213 treated with vancomycin (0.25–32 mg/L) and without as a positive control (*POS CON*). **b** 2D picture of *S. aureus* ATCC29213 obtained by the oCelloScope system, with a magnitude comparable to a 200× magnification in a standard light microscope

The oCelloScope is a digital time-lapse microscopy technology that scans through a fluid sample generating series of images as described in 2013 by Fredborg et al.[15]. As a result of the tilted imaging plane, the images recorded by the oCelloScope system constitute a parallelepipedum that forms the image stack. The projected z-stack image of a single z-plane was generated by combining the tilted images. Prior to image acquisition, setting the focus manually was performed for each of the 96 wells, taking 10–15 min. Image acquisition was performed in one scan area in the centre of each well covering approximately 40 % of the well, which was scanned repeatedly every 15 min for 12 h. Figure 1a shows one image plane recorded for the *E. coli* QC reference strains used for the evaluation of the oCelloScope system. The oCelloScope instrument was placed inside an Innova 44 incubator (New Brunswick Scientific), allowing precise temperature regulation.

Time-lapse experiments, digital analysis and image processing were conducted by a custom automation script in MATLAB [MATLAB v 8.0.0.783 (R2012b), The MathWorks Inc., Natick, MA, USA, 2000]. Growth kinetics were determined by image stack processing based on contrast-based segmentation and extraction of surface area (SESA).

### Data analysis

Bacterial susceptibility data for the QC reference strains obtained by the oCelloScope system were compared to QC ranges reported by TREK Diagnostic Systems (version 2.0). The TREK QC ranges were identical to the ranges given by the CLSI (M100-S23, 2013), with penicillin against *S. aureus* ATCC 29213 being the only outlier (Table 1). For clinical and blood culture isolates, antimicrobial susceptibility data obtained by the oCelloScope system were compared to manually read Sensititre® plates. The results from manually read Sensititre® plates were regarded as the gold standard. The time-to-result was measured from when the oCelloScope initiated image recording; the bacterial inoculum had been in contact with antimicrobials for approximately 10 min at this time point.

**Table 1:** Correlation between the oCelloScope system and TREK QC ranges for antimicrobial susceptibility of QC reference strains

	Antimicrobial susceptibility expressed as MIC values: oCelloScope value (TREK Diagnostic Systems QC range)			
Antimicrobial agent	*E. coli* ATCC25922	*S. pneumoniae* ATCC 49619	*S. aureus* ATCC 29213	*E. faecalis* ATCC 29212
Amikacin	2 (0.5–4)	–	–	–
Ampicillin	–	–	–	1 (0.5–1)
Ampicillin/sulbactam 2:1 ratio	4/2 (2/1–8/4)	–	–	–
Aztreonam	≤0.12 (0.06–0.25)	–	–	–
Ceftazidime	0.25 (0.06–0.5)	–	–	–
Cefoperazone	≤0.25 (0.12–0.5)	–	–	–
Cefoxitin screen	–	–	≤6 (≤6)	–
Cefotaxime	≤0.25 (0.03–0.12)	≤0.25 (0.03–0.12)	–	–
Ceftazidime	0.25 (0.06–0.5)	–	–	–
Ceftriaxone	≤0.25 (0.03–0.12)	≤0.06 (0.03–0.12)	–	–
Chloramphenicol	4 (2–8)	1 (2–8)	8 (2–16)	4 (4–16)
Ciprofloxacin	≤0.06 (0.004–0.015)	–	≤0.25 (0.12–0.5)	0.5 (0.25–2)
Clindamycin	–	0.25 (0.03–0.12)	≤0.12 (0.06–0.25)	>2 (4–16)
Daptomycin	–	0.06 (0.06–0.5)	0.5 (0.12–1)	2 (1–4)
Erythromycin	–	≤0.12 (0.03–0.12)	0.25 (0.25–1)	1 (1–4)
Gentamicin	1 (0.25–1)	–	≤0.5 (0.12–1)	–
Imipenem	0.12 (0.06–0.25)	–	–	–
Levofloxacin	0.03 (0.008–0.06)	0.5 (0.5–2)	0.25 (0.06–0.5)	1 (0.25–2)
Linezolid	–	0.25 (0.25–2)	2 (1–4)	2 (1–4)
Moxifloxacin	–	≤0.5 (0.06–0.25)	≤0.06 (0.015–0.12)	0.12 (0.06–0.5)
Nitrofurantoin	–	–	16 (8–32)	16 (4–16)
Oxacillin	–	–	0.25 (0.12–0.5)	>4 (8–32)
Penicillin	–	0.25 (0.25–1)	0.25* (0.12–1)	2 (1–4)
Piperacillin	2 (1–4)	–	–	–
Piperacillin/tazobactam 4	2/4 (1/4–4/4)	–	–	–
Quinupristin/dalfopristin	–	–	0.25 (0.25–1)	2 (2–8)
Rifampin	–	–	≤0.12 (0.004–0.015)	0.5 (0.5–4)
Tetracycline	2 (0.5–2)	≤0.5 (0.06–0.5)	≤0.5 (0.12–1)	16 (8–32)
Ticarcillin	16 (4–16)	–	–	–
Ticarcillin/clavulanic acid 2	8/2 (4/2–16/2)	–	–	–
Tigecycline	–	0.06 (0.015–0.12)	0.06 (0.03–0.25)	0.12 (0.03–0.12)
Tobramycin	1 (0.25–1)	–	–	–
Trimethoprim/sulphamethoxazole	0.12/2.38 (≤0.5/9.5)	≤0.25/4.75 (0.12/2.4–1/19)	≤0.12/2.38 (≤0.5/9.5)	≤0.12/2.38 (≤0.5/9.5)
Vancomycin	–	≤0.25 (0.12–0.5)	1 (1–4)	2 (1–4)

–: the antimicrobial is not present on the TREK Sensititre® plates chosen for AST. ≤: MIC value is below the range tested. >: MIC value is above the range tested. *: MIC value is different from those recommended in CLSI M100

Essential agreement was defined as MIC agreement within +/− one log_2_. All clinical isolates were classified as S (susceptible), I (intermediate) or R (resistant) according to the CLSI breakpoints (M100-S23, 2013). Categorical agreement was defined as agreement within the S-I-R classification. Minor errors were defined as susceptible or resistant isolates misclassified as intermediate or intermediate isolates misclassified as resistant or susceptible, major errors were defined as susceptible isolates misclassified as resistant and very major errors were defined as resistant isolates misclassified as susceptible.

GraphPad Prism version 6.00 for Windows (GraphPad Software, San Diego, CA, USA) was used for data and statistical analysis, as well as graphing.

## Results

### Antimicrobial susceptibility testing of quality control (QC) reference strains

The ability of the oCelloScope system to determine antibiotic susceptibility was assessed by testing four QC reference strains, *E. coli* ATCC25922, *S. aureus* ATCC29213, *S. pneumoniae* ATCC49619 and *E. faecalis* ATCC29212, using the Sensititre® microdilution plates. Figure 1 shows the image output (a) and growth curves (b) determined by the oCelloScope system for *S. aureus* ATCC29213. Agreements and discrepancies between MIC values obtained by the oCelloScope system and target QC ranges given by TREK Diagnostic Systems are presented in Table 1. Due to the concentration ranges of the antimicrobial panels, it was not possible to determine an exact MIC value for some antimicrobials (e.g. cefotaxime, tetracycline and trimethoprim/sulphamethoxazole). These values are given as ≤ than the lowest concentration in the panel range (all wells without growth) or > than the highest concentration in the panel range (growth in all wells). A total of 72 bacteria–antibiotic combinations were analysed, resulting in 97 % agreement between the results obtained by the oCelloScope system and the TREK target QC range. Similar results were obtained in three separate identical experiments. The average time-to-result for QC reference strains by the oCelloScope system was 102 min, with 93 % of the results being available within 180 min (Table 2). Two discrepancies of the 72 bacteria–antibiotic combinations were observed: *S. pneumoniae* tested against chloramphenicol and clindamycin (Table 1). For chloramphenicol, the MIC value measured by the oCelloScope system was one log_2_ concentration lower than the QC range, whereas the MIC value for clindamycin was one log_2_ concentration higher than the QC range.

**Table 2:** Time-to-result in minutes required for the oCelloScope system to gain AST results for four QC (ATCC) strains, nine clinical isolates and three positive blood cultures (*BC*)

Strain	av. time	25th percentile	75th percentile	min	max
*S. aureus* ATCC	118	53	143	30	360
*E. coli* ATCC	93	49	120	30	180
*E. faecalis* ATCC	112	68	143	45	270
*S. pneumoniae* ATCC	84	60	90	30	165
*S. aureus* MRSA	75	30	90	30	240
*S. aureus* MSSA	86	45	120	45	180
*S. epidermidis* Pen-R	103	60	120	30	240
*S. epidermidis* Pen-S	92	60	120	30	180
*S. pneumoniae*	91	60	90	45	180
*E. coli* ESBL	113	60	155	45	300
*E. coli* Amp-R	117	60	120	30	390
*E. faecalis* VRE	130	60	120	30	600
*E. faecalis* Gen-R	94	45	180	30	180
*S. aureus* BC	248	230	230	230	450
*E. coli* BC	109	90	120	45	280
*E. faecalis* BC	62	45	75	30	105

Results are expressed as average time (*av. time*), 25th percentile, 75th percentile, minimum time-to-result (*min*) and maximum time-to-result (*max*)

### Antimicrobial susceptibility testing of clinical isolates

Nine clinical isolates representing five different bacterial species with distinctive resistance profiles were included in this study (see the section on the experimental procedures for further information). A total of 168 bacteria–antibiotic combinations were tested using the Sensititre® microdilution plates. MIC values obtained by the oCelloScope system were compared to MIC values obtained by manual reading of Sensititre® plates. The average time-to-result for bacterial isolates by the oCelloScope system was 100 min, with 95 % of the results being available ≤180 min (Table 2). The average essential and categorical agreement was 93 % (156 of 168) and 93 % (136 of 146), respectively. Bacteria–antibiotic combinations that were not in agreement with conventional methods are listed in Table 3. Two major errors (1.2 %) were observed: *S. epidermidis* tested against nitrofurantoin and *E. coli* tested against chloramphenicol. Five minor errors (3.6 %) were observed: one for *S. pneumoniae* and four for *E. faecalis*, where two isolates were classified as rifampicin susceptible, one isolate was classified as linezolid intermediate, one isolate was classified as vancomycin intermediate and one isolate was classified as erythromycin intermediate. There were no major errors for any of the clinical isolates.

**Table 3:** AST of nine clinical isolates by the oCelloScope system and compared to the manually read Sensititre® system (gold standard)

Organism	No. of antimicrobials tested (antimicrobials with CLSI breakpoint)	Essential agreement ±1 log_2_ (%)	Categorical agreement (S-I-R according to CLSI breakpoints)			
			%	Minor errors	Major errors	Very major errors
*S. epidermidis*	19 (17)	90	97	0	0	1^a^
*S. pneumoniae*	20 (18)	85	94	1^b^	0	0
*S. aureus*	19 (17)	100	100	0	0	0
*E. faecalis*	15 (11)	83	78	5^c^	0	0
*E. coli*	22 (19)	100	98	0	0	1^d^

Results are expressed as essential agreements ±1 log_2_ and categorical agreements according to CLSI breakpoints

^a^ *S. epidermidis*: one isolate classified as nitrofurantoin susceptible

^b^ *S. pneumoniae*: one isolate classified as penicillin susceptible

^c^ *E. faecalis*: two isolates classified as rifampicin susceptible, one isolate classified as linezolid intermediate, one isolate classified as vancomycin intermediate and one isolate classified as erythromycin intermediate

^d^ *E. coli*: one isolate classified as chloramphenicol susceptible

Strains with MRSA and ESBL resistance profiles were detected correctly by the oCelloScope system. The vancomycin-resistant enterococci (VRE) strain was detected with a minor error regarding vancomycin: oCelloScope MIC = 8 mg/L vs. Sensititre® MIC >32 mg/L.

### Antimicrobial susceptibility testing on positive blood cultures

To evaluate the ability of the oCelloScope system to perform AST, three positive blood cultures were included. The selection of appropriate antimicrobial Sensititre® panels and growth media was based on morphology and Gram staining. Species identification of the bacteria was performed at Aarhus University Hospital and the results were not available prior to AST by the oCelloScope system. Table 4 shows the AST results obtained by the oCelloScope system compared to susceptibility results obtained by testing the positive blood cultures with the manual Sensititre® system. The average time-to-result for susceptibility testing by the oCelloScope system ranged from 1 to 4.2 h, depending on the bacteria–antibiotic combination and whether the bacteria had reached stationary growth phase prior to testing (Table 2). The overall essential agreement was 95 % (57 of 60). Two of the discrepancies were *S. aureus* tested against ampicillin (oCelloScope MIC = 1 mg/L vs. Sensititre® MIC = 4 mg/L) and *S. aureus* tested against penicillin (oCelloScope MIC = 0.5 mg/L vs. Sensititre® MIC = 4 mg/L). Both methods categorised the *S. aureus* isolate as resistant to penicillin. The third discrepancy was observed for *E. coli* tested against chloramphenicol (oCelloScope MIC 8 mg/L vs. Sensititre® MIC > 16 mg/L). The categorical agreement was 100 % for *S. aureus* and *E. faecalis*.

**Table 4:** Results of AST of positive blood cultures. Positive blood cultures analysed with the oCelloScope system compared to MIC values obtained by testing the blood culture isolates with the manually read Sensititre® system

	oCelloScope MIC value (Sensititre® MIC value)		
Antimicrobial agent	*E. coli*	*S. aureus*	*E. faecalis*
Amikacin	≤4 (≤4)	–	–
Ampicillin	–	1 (4)	1 (1)
Ampicillin/sulbactam 2:1 ratio	≤2/1 (≤2/1)	–	–
Aztreonam	≤2 (≤2)	–	–
Carbenicillin	≤32 (≤32)	–	–
Ceftazidime	≤1 (≤1)	–	–
Cefepime	≤2 (≤2)	–	–
Cefoperazone	≤4 (≤4)	–	–
Cefotaxime	≤4 (≤4)	–	–
Ceftriaxone	≤4 (≤4)	–	–
Chloramphenicol	16 (8)	4 (8)	8 (8)
Ciprofloxacin	≤0.25 (≤0.25)	≤1 (≤1)	≤1 (≤1)
Clindamycin	–	≤0.5 (≤0.5)	>2 (>2)
Daptomycin	–	≤0.5 (≤0.5)	1 (1)
Erythromycin	–	≤0.25 (0.5)	2 (2)
Gentamicin	≤1 (≤1)	≤2 (≤2)	16 (16)
Imipenem	≤1 (≤1)	–	–
Levofloxacin	≤0.12 (≤0.12)	≤0.25 (≤0.25)	1 (1)
Linezolid	–	≤1 (2)	2 (2)
Lomefloxacin	≤0.5 (≤0.5)	–	–
Moxifloxacin	–	≤0.25 (≤0.25)	≤0.25 (≤0.25)
Nitrofurantoin	–	≤32 (≤32)	≤32 (≤32)
Oxacillin	–	≤0.25 (≤0.25)	>4 (>4)
Penicillin	–	0.5 (4)	8 (4)
Piperacillin	≤8 (≤8)	–	–
Piperacillin/tazobactam 4	≤8/4 (≤8/4)	–	–
Quinupristin/dalfopristin	–	≤0.5 (≤0.5)	>4 (>4)
Rifampin	–	≤0.5 (≤0.5)	2 (2)
Tetracycline	≤1 (≤1)	≤2 (≤2)	>16 (>16)
Ticarcillin	≤8 (≤8)	–	–
Ticarcillin/clavulanic acid 2	≤16/2 (≤16/2)	–	–
Tigecycline	–	0.12 (0.06)	0.25 (0.12)
Tobramycin	≤1 (≤1)	–	–
Trimethoprim/sulphamethoxazole	≤0.5/9.5 (≤0.5/9.5)	≤0.5/9.5 (≤0.5/9.5)	≤0.5/9.5 (≤0.5/9.5)
Vancomycin	–	1 (1)	2 (2)

–: the antimicrobial was not included on the chosen TREK Sensititre® plate. ≤: MIC value is below the range tested. >: MIC value is above the range tested

## Discussion

In this study, we performed an initial screening to evaluate the potential of the oCelloScope system to perform rapid AST. This initial screening constitutes proof-of-concept in relation to bloodstream infections and, although this is not a full validation of the oCelloScope system, the results are compared to commercially available systems.

Four QC reference strains were used to evaluate the ability of the oCelloScope system for susceptibility determination, as directed by standards for the assessment of antimicrobial susceptibility [16, 17], with 95 % of the results lying within the QC target range. Subsequently, the evaluation was extended to include 168 bacteria–antibiotic combinations of common blood culture pathogens with different resistance profiles, resulting in an overall agreement of 96 %. It has been reported that an overall error rate of <10 % should be obtainable for an accepted performance of susceptibility testing [18–20]. According to guidelines, categorical agreements should be >90 %, major errors should be <3 % and very major errors should be <1.5 % [21]. In this study, the AST results of bacteria–antibiotic combinations with CLSI defined breakpoints showed categorical agreement for 93 %, 1.2 % very major errors and no major errors when determined by the oCelloScope system.

The AST results obtained in the two multidrug-resistant organisms, an MRSA and an ESBL-producing *E. coli*, showed no discrepancies in categorical agreement. Evaluation of the Vitek 2 and Phoenix systems for AST on blood cultures demonstrated categorical agreement rates of 94.6 % for Vitek 2 and 100 % for Phoenix when analysing 20 methicillin-resistant staphylococci tested against five antimicrobial agents [13]. Categorical agreements for 23 ESBL-positive *E. coli* tested against three antimicrobial agents were 97.5 % and 98.1 % for Vitek 2 and Phoenix, respectively [13]. For the VRE strain, oCelloScope determined it as intermediate, while manual reading classified it as resistant to vancomycin. The VRE strain carried the *vanB* gene. The *vanB* genotype usually has a vancomycin MIC distribution in the range 8–32 mg/L and generally confers lower MIC to vancomycin than the *vanA* genotype (typical vancomycin MIC > 64 mg/L) [22]. Rapid detection of the *vanB* genotype has proven to be difficult in automated systems and has led to the development of optimised panels [23, 24]. The disc diffusion test, agar screen and Etest showed 93 %, 100 % and 100 % sensitivity for detecting the *vanB* genotype, respectively, although agar diffusion tests can be challenging, as they require careful assessment of zone edges (eucast.org, CLSI M100 [23]. Thus, a full validation of the oCelloScope system should include a panel of multidrug-resistant species to determine its potential to rapidly determine the resistance profiles of such strains.

VME were observed with nitrofurantoin for *S. epidermidis* and with chloramphenicol for *E. coli*, both categorised as susceptible by the oCelloScope system. In a previous study evaluating Vitek 2, essential agreements with nitrofurantoin were 93.7 % for staphylococci and 88.9 % for enterococci [25], indicating that susceptibility testing for nitrofurantoin may be difficult for automated instruments. A minor error was observed for *E. faecalis* when tested against linezolid. Manual reading classified the isolate as susceptible (MIC values ≤1 mg/L and 2 mg/L), while the oCelloScope system classified both isolates as intermediate (MIC = 4 mg/L for both isolates). This discrepancy may be explained by the fact that linezolid MIC is determined at 80–90 % inhibition of growth (i.e. visible growth in negative wells) for manual reading, while the oCelloScope system measured MIC at 100 % inhibition of growth. The oCelloScope software, however, can be adjusted to allow for 10–20 % growth. For all other discrepancies, measurements performed by the oCelloScope system resulted in lower MIC values than the manually read Sensititre® plates, which were expected, since rapid AST methods generally result in lower MIC values.

Based on the promising results from analysis of the clinical isolates, the ability of the oCelloScope system to analyse positive blood cultures was examined. Only one discrepancy was observed in the 60 bacteria–antibiotic combinations: *E. coli* against chloramphenicol. Two possible sources of errors in AST are direct inoculation of mixed cultures and non-standardised inoculum size [26–28]. In some cases, mixed blood cultures can be determined by microscopy and Gram staining and, thus, be excluded from the AST method. If the blood culture contains bacteria with different morphology, this would be visible in the oCelloScope system and precautions may be taken. In order to ensure standardised inoculum size, all bacterial cell suspensions were adjusted to McFarland 0.5. Although blood cells and traces of blood culture broth can interfere with measurements, this would be visible in the oCelloScope system when setting up the experiment. For many compounds, the MIC value increases as inoculum increases (e.g. *S. aureus* tested against ciprofloxacin, oxacillin and vancomycin), while other antimicrobials such as chloramphenicol or the tetracyclines are not associated with inoculum effects [29, 30]. An advantage of the oCelloScope system, as well as other growth-based susceptibility tests, is that it only takes live bacteria into account and that this method, in contrast to agar diffusion tests, is independent of the antibiotic diffusion rate and time to macroscopically visible growth. Taken together, the results from studies on QC reference strains, clinical isolates and positive blood cultures showed an overall high accuracy of the oCelloScope system.

A major aim of this study was to assess whether the oCelloScope system could reduce the turnaround time of AST compared to conventional AST methods. If so, the oCelloScope system might serve as an essential screening tool to accelerate appropriate antibiotic therapy. The benefits of timely reporting of susceptibility results have been highlighted previously [31, 32]. Testing of QC reference strains and clinical isolates resulted in an average time-to-result of 100 min, with MIC determination being possible for 43 % of the bacteria–antibiotic combinations within 1 h (Table 2). However, it is possible to decrease the time-to-result even further by optimising algorithms and software settings, as the oCelloScope system was set up to analyse every 15 min for 12 h. Naturally, if the oCelloScope system was set up to analyse every 5 min, the time-to-result would have been shorter. Furthermore, a 2-h culture step was included to ensure early to mid-exponential phase of growth of the bacteria prior to analysis by the oCelloScope system. In a clinical setting, this step could be omitted, as the technique of the oCelloScope system could be integrated into automated blood culturing systems and, therefore, perform AST directly on positive blood cultures.

For AST on the included positive blood cultures, the average time-to-result ranged from 1 to 4.5 h, depending on the bacteria–antibiotic combination and whether the bacteria had reached the stationary growth phase. All blood cultures used in this study became positive during the night and were not processed until 8 o’clock the following morning. Consequently, these cultures may have been positive for up to 13 h. A lag phase of 2 h was observed for the *S. aureus* blood culture, which is the major reason for the prolonged time-to-result compared to that of the QC reference strains and the clinical isolates. According to the manufacturer’s instructions, the incubation time of Sensititre® MIC plates should be 18 h. This means a reduction in the average time-to-result of approximately 16.5 h by using the oCelloScope system. The fastest commercially available automatic AST method (Vitek 2, bioMeriéux, France) requires approximately 8 h to detect antimicrobial resistances in bacteria [33]. Taken together, these results strongly indicate that the oCelloScope system has the potential to decrease the time-to-result for AST. Translating this into daily workflow entails that same-day treatment or adjustments of antibiotic therapy are possible with the oCelloScope system. Only a few methods enable AST under 1 h, and only one method enables the determination of a real MIC value; however, this method is currently not commercially available [7]. AST of blood cultures by oCelloScope using Sensititre® microdilution plates may be incorporated into the clinical laboratory workflow, with 95 % of the results being available within 3 h and can be confirmed by inspection of the movies provided for each of the 96 wells.

The results from the three different types of samples clearly show that several parameters influence the time-to-result, including bacterial growth phase, bacterial doubling time, mode-of-action of the antibiotic and bacterial expression of resistance genes. However, it is clear that the prolonged time-to-result for the positive blood cultures is due to the growth state of the bacteria. Large variations of the time-to-result were observed for all samples, depending on the antibiotic of concern. As the number of isolates and species tested are limited, these variations might evolve when a greater number are included. However, it is crucial to note that part of these variations may not be clinically relevant, due to, for example, the site of infection, expression of resistance genes or antibiotic preference.

To take full advantages of the oCelloScope technique in a clinical setting, it should be incorporated into a fully automatic system, such as the blood culture system, or further developed into a fully automatic AST system of its own, and, most importantly, be able to analyse the bacterial infection as early as possible. However, considering the current version of the oCelloScope system, analysing a 96-well plate of growth-optimised bacteria using autofocus, continuing image acquisition and automated data analysis, the total time-to-result of AST would be around 60–75 min for 90 % of the analysed antimicrobials. The flexibility of the software makes adaption possible to most laboratory information management systems, even with the integration of movies from each well to ensure confirmation and acceptance of the test results by clinicians. The time-to-result for bacterial identification has been shortened considerably by the use of mass spectrometry techniques, and, therefore, no longer represents the bottleneck of AST. Consequently, the identification of a given infection is usually available before the results of AST and this information can, therefore, be incorporated into the data analysis part of the software.

## Conclusion

Although our data are preliminary, this study suggests that the oCelloScope system can decrease detection time, making same-day reporting possible and, thus, permitting better patient management. With an overall agreement of 96 % for the nine tested isolates and three positive blood cultures, the rapid detection time does not interfere with sensitivity. Strains with methicillin-resistant *Staphylococcus aureus* (MRSA) and extended-spectrum β-lactamase (ESBL) resistance profiles were rapidly detected by the oCelloScope system. A full-scale validation of the oCelloScope system including more isolates is necessary to assess the impact of using it for antimicrobial susceptibility testing (AST).
